# Correction: Game bird carcasses are less persistent than raptor carcasses, but can predict raptor persistence dynamics

**DOI:** 10.1371/journal.pone.0332044

**Published:** 2025-09-10

**Authors:** Eric Hallingstad, Daniel Riser-Espinoza, Samantha Brown, Paul Rabie, Jeanette Haddock, Karl Kosciuch

The images for Figs 6 and 7 are incorrectly switched. The image that appears as Fig 6 should be Fig 7, and the image that appears as Fig 7 should be Fig 6. The figure captions appear in the correct order. The authors have provided a corrected version of figures here.

**Fig 6:**
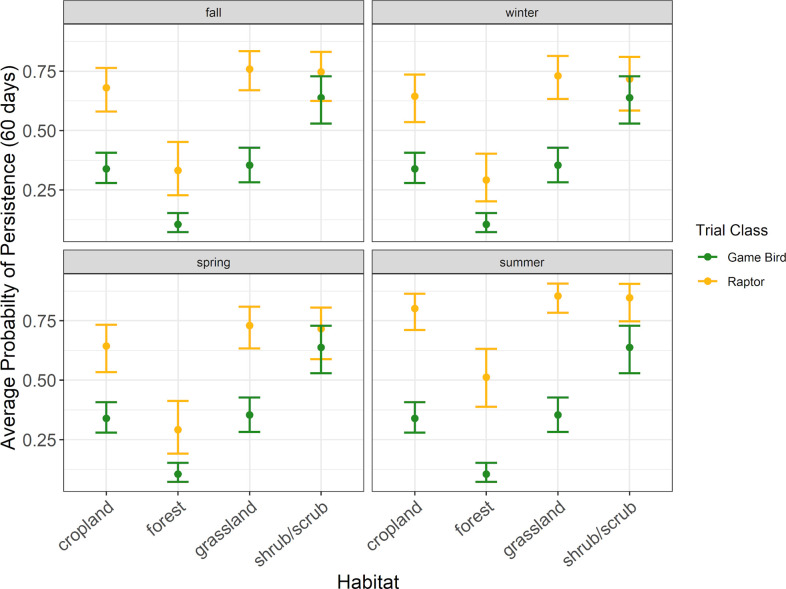
The average probability a raptor or game bird carcass would persist for 60 days in 4 habitat types during the carcass persistence study conducted June 2020 –August 2021.

**Fig 7:**
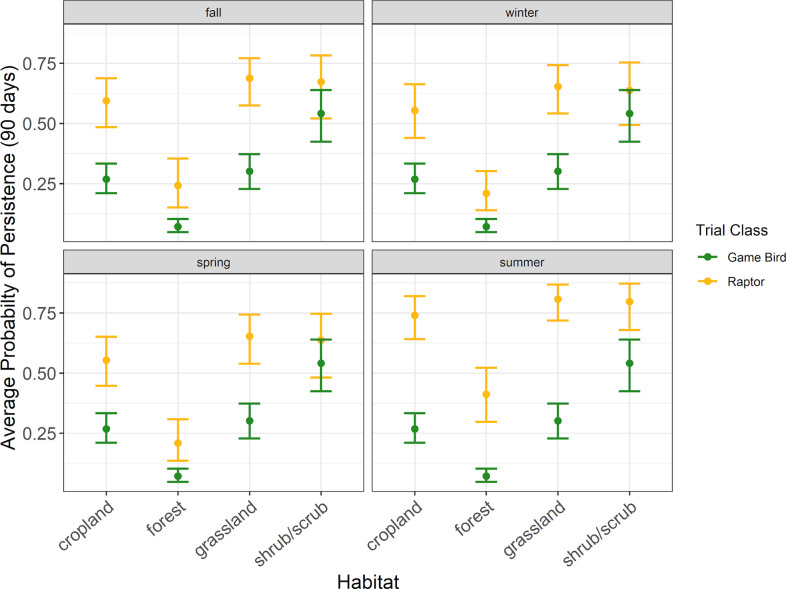
The average probability a raptor or game bird carcass would persist for 90 days in 4 habitat types during the carcass persistence study conducted June 2020 –August 2021.
