# Supplementary material for: Game bird carcasses are less persistent than raptor carcasses, but can predict raptor persistence dynamics
Source: PLoS One. 2023 Jan 3;18(1):e0279997. doi: 10.1371/journal.pone.0279997 (PMC9810176; doi:10.1371/journal.pone.0279997)
Supplement: S4 Table — Median persistence times and probabilities of persistence are for 3 search intervals (SIs; 30 days, 60 days, and 90 days), with 90% confidence intervals (CIs), for the carcass persistence study conducted from June 2020 –August 2021. (DOCX) [file pone.0279997.s004.docx]

**S4 Table. Estimates of median raptor carcass persistence times (in days) and average probabilities of persistence.** Median persistence times and probabilities of persistence are for 3 search intervals (SIs; 30 days, 60 days, and 90 days), with 90% confidence intervals (CIs), for the carcass persistence study conducted from June 2020 – August 2021.

| **Habitat** | **Season** | **Number of Trials** | **Median Persistence Time in Days (90%CI)** | **Average Probability of Persistence, SI = 30 Days (90% CI)** | **Average Probability of Persistence, SI = 60 Days (90% CI)** | **Average Probability of Persistence, SI = 90 Days (90% CI)** |
| --- | --- | --- | --- | --- | --- | --- |
| cropland | fall | 20 | 56.7 (35.6–88.4) | 0.80 (0.71–0.86) | 0.68 (0.58–0.76) | 0.59 (0.48–0.69) |
| cropland | spring | 20 | 47.5 (29.1–76.3) | 0.77 (0.68–0.84) | 0.64 (0.53–0.73) | 0.55 (0.45–0.65) |
| cropland | summer | 20 | 118.4 (66.4–198.1) | 0.88 (0.81–0.92) | 0.80 (0.71–0.86) | 0.74 (0.64–0.82) |
| cropland | winter | 20 | 47.6 (29.2–77.8) | 0.77 (0.68–0.84) | 0.64 (0.54–0.74) | 0.55 (0.44–0.66) |
| forest | fall | 10 | 12.7 (7.5–20.9) | 0.50 (0.37–0.62) | 0.33 (0.23–0.45) | 0.24 (0.15–0.35) |
| forest | spring | 10 | 10.7 (6.1–17.8) | 0.46 (0.33–0.58) | 0.29 (0.19–0.41) | 0.21 (0.14–0.31) |
| forest | summer | 10 | 26.6 (16.2–42.7) | 0.67 (0.56–0.76) | 0.51 (0.39–0.63) | 0.41 (0.30–0.52) |
| forest | winter | 10 | 10.7 (6.8–17.4) | 0.46 (0.35–0.58) | 0.29 (0.20–0.40) | 0.21 (0.14–0.30) |
| grassland | fall | 20 | 88.5 (51.4–154.4) | 0.85 (0.78–0.90) | 0.76 (0.67–0.83) | 0.69 (0.58–0.77) |
| grassland | spring | 20 | 74.3 (44.5–128.0) | 0.83 (0.76–0.88) | 0.73 (0.63–0.81) | 0.65 (0.54–0.74) |
| grassland | summer | 20 | 184.9 (103.3–333.5) | 0.91 (0.86–0.94) | 0.85 (0.78–0.91) | 0.81 (0.72–0.87) |
| grassland | winter | 20 | 74.4 (45.4–125.1) | 0.83 (0.76–0.89) | 0.73 (0.63–0.81) | 0.65 (0.54–0.74) |
| shrub/scrub | fall | 9 | 82.1 (40.8–157.2) | 0.84 (0.75–0.90) | 0.75 (0.62–0.83) | 0.67 (0.52–0.78) |
| shrub/scrub | spring | 10 | 68.9 (36.7–130.0) | 0.82 (0.72–0.89) | 0.72 (0.59–0.81) | 0.64 (0.48–0.75) |
| shrub/scrub | summer | 10 | 171.6 (81.5–346.2) | 0.91 (0.84–0.95) | 0.85 (0.75–0.91) | 0.80 (0.68–0.87) |
| shrub/scrub | winter | 10 | 69.0 (38.1–139.2) | 0.82 (0.73–0.89) | 0.72 (0.58–0.81) | 0.64 (0.49–0.75) |
