# Supplementary material for: Game bird carcasses are less persistent than raptor carcasses, but can predict raptor persistence dynamics
Source: PLoS One. 2023 Jan 3;18(1):e0279997. doi: 10.1371/journal.pone.0279997 (PMC9810176; doi:10.1371/journal.pone.0279997)
Supplement: S5 Table — Model selection used corrected Akaike’s Information Criterion (AICc) with seasonal and habitat covariates for the 240 large game bird carcasses placed during the carcass persistence study conducted June 2020 –August 2021. (DOCX) [file pone.0279997.s005.docx]

**S5 Table. Game bird persistence model selection.** Model selection used corrected Akaike’s Information Criterion (AICc) with seasonal and habitat covariates for the 240 large game bird carcasses placed during the carcass persistence study conducted June 2020 – August 2021.

| **Distribution** | **Location Parameter** | **Scale Parameter** | **Number of Parameters** | **AICc** | **Δ AICc** |
| --- | --- | --- | --- | --- | --- |
| lognormal | l ~ habitat | s ~ habitat | 8 | 979.02 | 0^a^ |
| loglogistic | l ~ habitat | s ~ habitat | 8 | 980.96 | 1.94 |
| lognormal | l ~ Season + habitat | s ~ habitat | 11 | 983.48 | 4.46 |
| lognormal | l ~ habitat | s ~ Season + habitat | 11 | 984.04 | 5.02 |
| loglogistic | l ~ Season + habitat | s ~ habitat | 11 | 985.98 | 6.96 |
| loglogistic | l ~ habitat | s ~ Season + habitat | 11 | 986.02 | 7.00 |
| lognormal | l ~ habitat | s ~ 1 | 5 | 987.48 | 8.46 |
| lognormal | l ~ Season + habitat | s ~ Season + habitat | 14 | 988.64 | 9.62 |

Models with Δ AICc (difference in AIC points from top model) less than or equal to 10 are shown above.

^a^ We used this model in the analysis.
