# Supplementary material for: Game bird carcasses are less persistent than raptor carcasses, but can predict raptor persistence dynamics
Source: PLoS One. 2023 Jan 3;18(1):e0279997. doi: 10.1371/journal.pone.0279997 (PMC9810176; doi:10.1371/journal.pone.0279997)
Supplement: S6 Table — Median persistence times and probabilities of persistence are for 3 search intervals (SIs; 30 days, 60 days, and 90 days), and 90% confidence intervals (CIs), for the carcass persistence study conducted from June 2020 –August 2021. (DOCX) [file pone.0279997.s006.docx]

**S6 Table. Estimates of median game bird persistence times (in days) and average probabilities of persistence.** Median persistence times and probabilities of persistence are for 3 search intervals (SIs; 30 days, 60 days, and 90 days), and 90% confidence intervals (CIs), for the carcass persistence study conducted from June 2020 – August 2021.

| **Habitat** | **Number of Trials** | **Median Persistence Time in Days (90%CI)** | **Average Probability of Persistence, SI = 30 Days (90% CI)** | **Average Probability of Persistence, SI = 60 Days (90% CI)** | **Average Probability of Persistence, SI = 90 Days (90% CI)** |
| --- | --- | --- | --- | --- | --- |
| cropland | 80 | 10.5 (7.5–14.8) | 0.48 (0.41–0.55) | 0.34 (0.28–0.41) | 0.27 (0.21–0.33) |
| forest | 40 | 3.2 (2.2–4.5) | 0.19 (0.14–0.26) | 0.11 (0.07–0.15) | 0.07 (0.05–0.10) |
| grassland | 79 | 8.2 (4.8–14.0) | 0.45 (0.37–0.53) | 0.35 (0.28–0.43) | 0.30 (0.23–0.37) |
| shrub/scrub | 41 | 42.7 (28.0–65.2) | 0.78 (0.70–0.85) | 0.64 (0.53–0.73) | 0.54 (0.42–0.64) |
