# Supplementary material for: Game bird carcasses are less persistent than raptor carcasses, but can predict raptor persistence dynamics
Source: PLoS One. 2023 Jan 3;18(1):e0279997. doi: 10.1371/journal.pone.0279997 (PMC9810176; doi:10.1371/journal.pone.0279997)
Supplement: S7 Table — Model selection used corrected Akaike’s Information Criterion (AICc) with seasonal, habitat, and USFWS Region covariates for the meta-dataset collected for the carcass persistence study. (DOCX) [file pone.0279997.s007.docx]

**S7 Table. Raptor persistence model selection.** Model selection used corrected Akaike’s Information Criterion (AICc) with seasonal, habitat, and USFWS Region covariates for the meta-dataset collected for the carcass persistence study.

| **Distribution** | **Location Parameter** | **Scale Parameter** | **Number of Parameters** | **Sample Size** | **AICc** | **Δ AICc** |
| --- | --- | --- | --- | --- | --- | --- |
| Weibull | l ~ Season + Habitat + Region + Season:Region | s ~ Season + Region | 41 | 1,624 | 5826.77 | 0^a^ |
| Weibull | l ~ Season + Habitat + Region + Season:Region | s ~ Region | 38 | 1,624 | 5829.72 | 2.95 |
| Weibull | l ~ Season + Region + Season: Region | s ~ Season + Habitat + Region + Season:Habitat | 50 | 1,624 | 5829.74 | 2.97 |
| Weibull | l ~ Season + Region + Season: Region | s ~ Season + Habitat + Region | 41 | 1,624 | 5831.34 | 4.57 |
| Weibull | l ~ Season +Region + Season: Region | s ~ Season + Region | 38 | 1,624 | 5831.41 | 4.64 |
| Weibull | l ~ Season + Habitat + Region + Season: Region | s ~ Season + Habitat + Region | 44 | 1,624 | 5831.91 | 5.14 |
| Weibull | l ~ Season + Habitat + Region + Season: Region | s ~ Season + Habitat + Region + Season:Habitat | 53 | 1,624 | 5831.93 | 5.16 |
| Weibull | l ~ Season + Region + Season: Region | s ~ Season + Habitat | 35 | 1,624 | 5833.35 | 6.58 |
| Weibull | l ~ Season + Region + Season: Region | s ~ Region | 35 | 1,624 | 5833.39 | 6.62 |
| Weibull | l ~ Season + Habitat + Region + Season: Region | s ~ Habitat + Region | 41 | 1,624 | 5834.50 | 7.73 |
| loglogistic | l ~ Season + Habitat + Region + Season: Region | s ~ Region | 38 | 1,624 | 5834.58 | 7.81 |
| Weibull | l ~ Season + Region + Season: Region | s ~ Habitat + Region | 38 | 1,624 | 5834.94 | 8.17 |
| Weibull | l ~ Season + Habitat + Region + Season: Region | s ~ Season + Habitat | 38 | 1,624 | 5834.98 | 8.21 |
| loglogistic | l ~ Season + Habitat + Region + Season: Region | s ~ Season + Region | 41 | 1,624 | 5835.20 | 8.43 |

Models with Δ AICc (difference in AIC points from top model) less than or equal to 10 are shown above.

^a^ We used this model in the analysis.
