# Supplementary material for: Game bird carcasses are less persistent than raptor carcasses, but can predict raptor persistence dynamics
Source: PLoS One. 2023 Jan 3;18(1):e0279997. doi: 10.1371/journal.pone.0279997 (PMC9810176; doi:10.1371/journal.pone.0279997)
Supplement: S8 Table — Model selection used corrected Akaike’s Information Criterion (AICc) with seasonal, habitat, and USFWS Region covariates for the meta-dataset collected for the carcass persistence study. (DOCX) [file pone.0279997.s008.docx]

**S8 Table. Game bird persistence model selection.** Model selection used corrected Akaike’s Information Criterion (AICc) with seasonal, habitat, and USFWS Region covariates for the meta-dataset collected for the carcass persistence study.

| **Distribution** | **Location Parameter** | **Scale Parameter** | **Number of Parameters** | **Sample Size** | **AICc** | **Δ AICc** |
| --- | --- | --- | --- | --- | --- | --- |
| loglogistic | l ~ Season + Habitat + Region + Habitat:Region | s ~ Region | 38 | 1,747 | 7582.45 | 0^a^ |
| loglogistic | l ~ Season + Habitat + Region + Season:Habitat + Habitat: Region | s ~ Region | 47 | 1,747 | 7586.85 | 4.40 |
| loglogistic | l ~ Season + Habitat + Region + Habitat: Region | s ~ Habitat | 35 | 1,747 | 7589.23 | 6.78 |

Models with Δ AICc (difference in AIC points from top model) less than or equal to 10 are shown above.

^a^ We used this model in the analysis.
