# Supplementary material for: Game bird carcasses are less persistent than raptor carcasses, but can predict raptor persistence dynamics
Source: PLoS One. 2023 Jan 3;18(1):e0279997. doi: 10.1371/journal.pone.0279997 (PMC9810176; doi:10.1371/journal.pone.0279997)
Supplement: S9 Table — Median persistence times and average probabilities of persistence are by USFWS Region, habitat, and season for 3 search intervals (30 days, 60 days, and 90 days), with 90% confidence intervals (CIs). (DOCX) [file pone.0279997.s009.docx]

**S9 Table. Estimates of median raptor persistence times and average probabilities of persistence.** Median persistence times and average probabilities of persistence are by USFWS Region, habitat, and season for 3 search intervals (30 days, 60 days, and 90 days), with 90% confidence intervals (CIs).

| **Region** | **Habitat** | **Season** | **Number of Trials** | **Median Persistence Time in Days (90%CI)** | **Average Probability of Persistence, SI = 30 Days (90% CI)** | **Average Probability of Persistence, SI = 60 Days (90% CI)** | **Average Probability of Persistence, SI = 90 Days (90% CI)** |
| --- | --- | --- | --- | --- | --- | --- | --- |
| 1 | cropland | fall | 29 | 39.5 (24.9–61.7) | 0.71 (0.63–0.78) | 0.59 (0.5–0.68) | 0.51 (0.42–0.6) |
| 1 | cropland | spring | 32 | 202.1 (121.1–327.1) | 0.92 (0.88–0.95) | 0.86 (0.8–0.91) | 0.82 (0.75–0.87) |
| 1 | cropland | summer | 6 | 108 (68.6–177.3) | 0.89 (0.83–0.93) | 0.81 (0.71–0.88) | 0.74 (0.63–0.83) |
| 1 | cropland | winter | 41 | 48.9 (33.1–72.9) | 0.75 (0.69–0.81) | 0.64 (0.56–0.71) | 0.56 (0.48–0.64) |
| 1 | forest | summer | 13 | 51.1 (26.9–95.2) | 0.8 (0.68–0.88) | 0.67 (0.52–0.79) | 0.57 (0.41–0.72) |
| 1 | grassland | fall | 5 | 23.2 (14.6–37.1) | 0.62 (0.53–0.71) | 0.48 (0.38–0.58) | 0.4 (0.3–0.5) |
| 1 | grassland | spring | 12 | 118.6 (70.1–199.8) | 0.88 (0.82–0.92) | 0.8 (0.71–0.86) | 0.74 (0.65–0.82) |
| 1 | grassland | summer | 42 | 63.4 (45.3–88) | 0.83 (0.77–0.88) | 0.72 (0.64–0.79) | 0.63 (0.54–0.71) |
| 1 | grassland | winter | 14 | 28.7 (18.6–44.3) | 0.67 (0.58–0.74) | 0.53 (0.43–0.62) | 0.44 (0.34–0.54) |
| 1 | shrub/scrub | fall | 20 | 40.7 (25.8–64.9) | 0.72 (0.64–0.79) | 0.6 (0.51–0.69) | 0.52 (0.42–0.61) |
| 1 | shrub/scrub | spring | 18 | 208.4 (121.7–344.4) | 0.92 (0.88–0.95) | 0.87 (0.81–0.91) | 0.82 (0.75–0.88) |
| 1 | shrub/scrub | winter | 20 | 50.5 (33–77.8) | 0.76 (0.68–0.82) | 0.64 (0.56–0.72) | 0.56 (0.47–0.65) |
| 2 | cropland | fall | 10 | 122.1 (56.6–257.6) | 0.85 (0.77–0.91) | 0.78 (0.66–0.86) | 0.72 (0.59–0.82) |
| 2 | cropland | spring | 10 | 101.2 (50.8–191.8) | 0.86 (0.78–0.92) | 0.78 (0.67–0.86) | 0.71 (0.57–0.81) |
| 2 | cropland | summer | 10 | 144.8 (70.6–297.4) | 0.91 (0.84–0.95) | 0.85 (0.73–0.91) | 0.79 (0.65–0.88) |
| 2 | cropland | winter | 10 | 49.5 (30.3–79.7) | 0.75 (0.67–0.82) | 0.64 (0.54–0.72) | 0.56 (0.46–0.65) |
| 2 | grassland | fall | 21 | 71.7 (33.9–143.4) | 0.8 (0.69–0.86) | 0.7 (0.58–0.8) | 0.63 (0.49–0.74) |
| 2 | grassland | spring | 25 | 59.4 (31.9–103.4) | 0.8 (0.71–0.87) | 0.69 (0.57–0.78) | 0.61 (0.47–0.71) |
| 2 | grassland | summer | 16 | 85.0 (43.9–167.3) | 0.86 (0.78–0.92) | 0.77 (0.63–0.85) | 0.69 (0.53–0.81) |
| 2 | grassland | winter | 81 | 29.0 (21.4–39.5) | 0.67 (0.61–0.73) | 0.53 (0.47–0.6) | 0.44 (0.38–0.51) |
| 2 | shrub/scrub | summer | 7 | 149.3 (73.7–302.6) | 0.91 (0.85–0.95) | 0.85 (0.74–0.91) | 0.79 (0.66–0.88) |
| 2 | shrub/scrub | winter | 1 | 51.1 (31.6–78.2) | 0.76 (0.67–0.82) | 0.65 (0.56–0.73) | 0.57 (0.47–0.65) |
| 3 | cropland | fall | 54 | 56.3 (38.2–82.0) | 0.8 (0.73–0.85) | 0.68 (0.6–0.75) | 0.59 (0.49–0.67) |
| 3 | cropland | spring | 195 | 38.7 (33.0–44.9) | 0.77 (0.73–0.8) | 0.61 (0.57–0.65) | 0.5 (0.45–0.54) |
| 3 | cropland | summer | 46 | 36.1 (27.6–47.3) | 0.77 (0.7–0.83) | 0.6 (0.51–0.67) | 0.47 (0.39–0.56) |
| 3 | cropland | winter | 302 | 24.7 (21.6–28.2) | 0.66 (0.63–0.69) | 0.49 (0.46–0.52) | 0.39 (0.35–0.42) |
| 4 | cropland | fall | 10 | 270.8 (26.8–2213.7) | 0.83 (0.61–0.94) | 0.78 (0.49–0.91) | 0.75 (0.45–0.89) |
| 4 | cropland | spring | 10 | 36.4 (9.7–122.8) | 0.67 (0.46–0.83) | 0.57 (0.33–0.75) | 0.5 (0.27–0.71) |
| 4 | cropland | summer | 10 | 49.5 (13.1–182.7) | 0.73 (0.51–0.87) | 0.62 (0.38–0.81) | 0.55 (0.29–0.76) |
| 4 | cropland | winter | 10 | 13.8 (2.8–53.7) | 0.52 (0.3–0.72) | 0.41 (0.19–0.64) | 0.35 (0.16–0.56) |
| 5 | forest | fall | 10 | 11.3 (6.7–19.3) | 0.45 (0.31–0.61) | 0.25 (0.16–0.39) | 0.17 (0.11–0.27) |
| 5 | forest | spring | 10 | 11.3 (7.0–18.9) | 0.44 (0.30–0.62) | 0.23 (0.15–0.35) | 0.16 (0.10–0.24) |
| 5 | forest | summer | 10 | 32.4 (22.2–47.8) | 0.79 (0.67–0.89) | 0.56 (0.43–0.69) | 0.41 (0.29–0.55) |
| 5 | forest | winter | 10 | 25.3 (13.9–44.1) | 0.69 (0.52–0.83) | 0.48 (0.31–0.66) | 0.36 (0.22–0.53) |
| 6 | cropland | fall | 1 | 113.5 (69.2–190.4) | 0.86 (0.80–0.91) | 0.78 (0.71–0.85) | 0.72 (0.62–0.79) |
| 6 | cropland | spring | 1 | 585.4 (322.6–1050.3) | 0.97 (0.95–0.99) | 0.95 (0.92–0.97) | 0.93 (0.89–0.96) |
| 6 | cropland | winter | 3 | 313.4 (183.5–558.4) | 0.94 (0.90–0.96) | 0.89 (0.85–0.93) | 0.86 (0.80–0.90) |
| 6 | grassland | fall | 42 | 66.6 (44.5–99.0) | 0.80 (0.74–0.86) | 0.70 (0.63–0.77) | 0.62 (0.54–0.69) |
| 6 | grassland | spring | 55 | 343.5 (208.2–539.2) | 0.96 (0.93–0.97) | 0.92 (0.88–0.95) | 0.89 (0.85–0.93) |
| 6 | grassland | summer | 55 | 210.0 (142.2–302.3) | 0.95 (0.92–0.97) | 0.90 (0.86–0.93) | 0.86 (0.81–0.90) |
| 6 | grassland | winter | 58 | 183.9 (123.9–280.5) | 0.91 (0.87–0.93) | 0.85 (0.80–0.88) | 0.80 (0.74–0.84) |
| 6 | shrub/scrub | fall | 44 | 117.2 (78.1–172.3) | 0.86 (0.82–0.90) | 0.79 (0.73–0.84) | 0.73 (0.66–0.78) |
| 6 | shrub/scrub | spring | 49 | 603.8 (345.8–982.7) | 0.97 (0.96–0.98) | 0.95 (0.92–0.97) | 0.93 (0.90–0.96) |
| 6 | shrub/scrub | summer | 46 | 369.1 (238.6–570.3) | 0.97 (0.95–0.98) | 0.94 (0.91–0.96) | 0.91 (0.87–0.94) |
| 6 | shrub/scrub | winter | 48 | 323.3 (206.0–500.2) | 0.94 (0.91–0.96) | 0.90 (0.86–0.93) | 0.86 (0.82–0.90) |
| 8 | shrub/scrub | fall | 18 | 73.2 (48.2–110.7) | 0.89 (0.82–0.95) | 0.78 (0.68–0.87) | 0.69 (0.56–0.79) |
| 8 | shrub/scrub | spring | 22 | 464.9 (180.6–1145.8) | 0.99 (0.98–1.00) | 0.98 (0.94–0.99) | 0.97 (0.90–0.99) |
| 8 | shrub/scrub | summer | 22 | 227.3 (127.3–414.8) | 0.99 (0.96–1.00) | 0.96 (0.91–0.99) | 0.93 (0.86–0.97) |
| 8 | shrub/scrub | winter | 40 | 56.0 (40.9–74.7) | 0.86 (0.79–0.91) | 0.73 (0.63–0.80) | 0.61 (0.50–0.7) |

All model results are based on the complete meta-dataset of persistence trials from the U.S.
