# Supplementary material for: Game bird carcasses are less persistent than raptor carcasses, but can predict raptor persistence dynamics
Source: PLoS One. 2023 Jan 3;18(1):e0279997. doi: 10.1371/journal.pone.0279997 (PMC9810176; doi:10.1371/journal.pone.0279997)
Supplement: S10 Table — Median persistence times and average probabilities of persistence are by USFWS Region, habitat, and season for 3 search intervals (30 days, 60 days, and 90 days), with 90% confidence intervals (CIs). (DOCX) [file pone.0279997.s010.docx]

**S10 Table. Estimates of median game bird persistence times and average probabilities of persistence.** Median persistence times and average probabilities of persistence are by USFWS Region, habitat, and season for 3 search intervals (30 days, 60 days, and 90 days), with 90% confidence intervals (CIs).

| **Region** | **Habitat** | **Season** | **Number of Trials** | **Median Persistence Time in Days (90%CI)** | **Average Probability of Persistence SI = 30 Days (90% CI)** | **Average Probability of Persistence, SI = 60 Days (90% CI)** | **Average Probability of Persistence, SI = 90 Days (90% CI)** |
| --- | --- | --- | --- | --- | --- | --- | --- |
| **1** | cropland | fall | 54 | 9.9 (8–12.3) | 0.45 (0.4–0.5) | 0.31 (0.27–0.35) | 0.24 (0.20–0.27) |
| **1** | cropland | spring | 49 | 14.9 (11.9–18.6) | 0.55 (0.49–0.6) | 0.39 (0.34–0.44) | 0.31 (0.27–0.36) |
| **1** | cropland | summer | 33 | 11.5 (9.2–14.4) | 0.49 (0.43–0.54) | 0.34 (0.29–0.39) | 0.26 (0.22–0.3) |
| **1** | cropland | winter | 61 | 15 (12.1–18.6) | 0.55 (0.5–0.6) | 0.39 (0.35–0.44) | 0.31 (0.27–0.35) |
| **1** | grassland | fall | 38 | 4.9 (3.9–6.2) | 0.31 (0.26–0.35) | 0.19 (0.16–0.23) | 0.14 (0.12–0.17) |
| **1** | grassland | spring | 5 | 7.4 (5.7–9.5) | 0.39 (0.33–0.45) | 0.25 (0.21–0.3) | 0.19 (0.16–0.23) |
| **1** | grassland | summer | 69 | 5.7 (4.6–7.2) | 0.34 (0.29–0.38) | 0.21 (0.18–0.25) | 0.16 (0.13–0.19) |
| **1** | grassland | winter | 32 | 7.4 (5.8–9.4) | 0.39 (0.34–0.44) | 0.26 (0.22–0.3) | 0.19 (0.16–0.23) |
| **1** | shrub/scrub | fall | 22 | 4.8 (3.4–6.7) | 0.30 (0.24–0.37) | 0.19 (0.15–0.24) | 0.14 (0.11–0.19) |
| **1** | shrub/scrub | spring | 22 | 7.2 (5.1–10.1) | 0.38 (0.31–0.46) | 0.25 (0.2–0.31) | 0.19 (0.15–0.25) |
| **1** | shrub/scrub | summer | 14 | 5.6 (3.9–7.9) | 0.33 (0.26–0.4) | 0.21 (0.16–0.27) | 0.16 (0.12–0.21) |
| **1** | shrub/scrub | winter | 5 | 7.2 (5.1–10.3) | 0.38 (0.31–0.46) | 0.25 (0.19–0.32) | 0.19 (0.15–0.25) |
| **2** | cropland | fall | 10 | 5.6 (3.5–8.7) | 0.34 (0.26–0.43) | 0.22 (0.17–0.3) | 0.17 (0.12–0.24) |
| **2** | cropland | spring | 10 | 8.3 (5.3–13.1) | 0.42 (0.33–0.52) | 0.29 (0.21–0.37) | 0.22 (0.17–0.3) |
| **2** | cropland | summer | 10 | 6.5 (4.1–10.1) | 0.37 (0.28–0.46) | 0.24 (0.18–0.32) | 0.19 (0.14–0.26) |
| **2** | cropland | winter | 10 | 8.4 (5.4–13.2) | 0.42 (0.33–0.52) | 0.29 (0.22–0.37) | 0.22 (0.17–0.3) |
| **2** | grassland | fall | 30 | 2.5 (1.9–3.2) | 0.20 (0.16–0.25) | 0.13 (0.1–0.16) | 0.09 (0.07–0.12) |
| **2** | grassland | spring | 28 | 3.7 (2.8–4.9) | 0.27 (0.22–0.32) | 0.17 (0.13–0.21) | 0.13 (0.1–0.16) |
| **2** | grassland | summer | 30 | 2.9 (2.2–3.8) | 0.23 (0.18–0.27) | 0.14 (0.11–0.18) | 0.1 (0.08–0.13) |
| **2** | grassland | winter | 30 | 3.7 (2.8–4.9) | 0.27 (0.22–0.32) | 0.17 (0.14–0.21) | 0.13 (0.1–0.16) |
| **2** | shrub/scrub | fall | 28 | 3.4 (2.4–5) | 0.25 (0.19–0.32) | 0.16 (0.12–0.21) | 0.12 (0.09–0.17) |
| **2** | shrub/scrub | spring | 6 | 5.2 (3.5–7.6) | 0.32 (0.25–0.4) | 0.21 (0.16–0.27) | 0.16 (0.12–0.22) |
| **2** | shrub/scrub | summer | 10 | 4 (2.7–5.9) | 0.28 (0.22–0.35) | 0.18 (0.13–0.23) | 0.13 (0.1–0.18) |
| **2** | shrub/scrub | winter | 15 | 5.2 (3.6–7.6) | 0.33 (0.26–0.4) | 0.21 (0.16–0.27) | 0.16 (0.12–0.22) |
| **3** | cropland | fall | 75 | 6.4 (5.4–7.6) | 0.34 (0.31–0.38) | 0.21 (0.18–0.24) | 0.15 (0.13–0.18) |
| **3** | cropland | spring | 121 | 9.7 (8.3–11.2) | 0.44 (0.4–0.48) | 0.28 (0.25–0.31) | 0.21 (0.18–0.24) |
| **3** | cropland | summer | 100 | 7.5 (6.4–8.8) | 0.38 (0.34–0.41) | 0.23 (0.21–0.26) | 0.17 (0.15–0.2) |
| **3** | cropland | winter | 314 | 9.8 (8.8–10.9) | 0.44 (0.41–0.47) | 0.28 (0.26–0.31) | 0.21 (0.19–0.23) |
| **4** | cropland | fall | 10 | 11.5 (6.2–21.1) | 0.5 (0.39–0.61) | 0.38 (0.28–0.48) | 0.31 (0.22–0.41) |
| **4** | cropland | spring | 10 | 17.2 (9.4–31.6) | 0.57 (0.47–0.68) | 0.45 (0.34–0.55) | 0.38 (0.28–0.48) |
| **4** | cropland | summer | 10 | 13.4 (7.3–24.6) | 0.52 (0.42–0.64) | 0.4 (0.3–0.51) | 0.34 (0.24–0.44) |
| **4** | cropland | winter | 10 | 17.4 (9.5–31.9) | 0.57 (0.47–0.69) | 0.45 (0.35–0.56) | 0.38 (0.28–0.48) |
| **5** | forest | fall | 10 | 2.6 (1.8–3.6) | 0.16 (0.11–0.22) | 0.09 (0.06–0.13) | 0.06 (0.04–0.09) |
| **5** | forest | spring | 10 | 3.8 (2.7–5.4) | 0.22 (0.16–0.29) | 0.13 (0.09–0.18) | 0.09 (0.06–0.13) |
| **5** | forest | summer | 10 | 3 (2.1–4.2) | 0.18 (0.13–0.25) | 0.1 (0.07–0.15) | 0.07 (0.05–0.11) |
| **5** | forest | winter | 10 | 3.9 (2.7–5.5) | 0.22 (0.16–0.3) | 0.13 (0.09–0.18) | 0.09 (0.06–0.13) |
| **6** | cropland | fall | 15 | 3.9 (2.7–5.5) | 0.27 (0.22–0.34) | 0.17 (0.13–0.23) | 0.13 (0.1–0.17) |
| **6** | cropland | spring | 15 | 5.8 (4.1–8.2) | 0.35 (0.28–0.42) | 0.23 (0.18–0.29) | 0.17 (0.13–0.22) |
| **6** | cropland | summer | 15 | 4.5 (3.2–6.4) | 0.3 (0.24–0.36) | 0.19 (0.15–0.25) | 0.14 (0.11–0.19) |
| **6** | cropland | winter | 15 | 5.9 (4.2–8.3) | 0.35 (0.29–0.42) | 0.23 (0.18–0.29) | 0.17 (0.13–0.22) |
| **6** | grassland | fall | 29 | 8.7 (6.4–11.7) | 0.43 (0.36–0.49) | 0.29 (0.24–0.35) | 0.23 (0.18–0.28) |
| **6** | grassland | spring | 33 | 13 (9.7–17.5) | 0.52 (0.45–0.58) | 0.37 (0.31–0.43) | 0.29 (0.24–0.35) |
| **6** | grassland | summer | 27 | 10.1 (7.5–13.6) | 0.46 (0.4–0.53) | 0.32 (0.26–0.38) | 0.25 (0.2–0.3) |
| **6** | grassland | winter | 24 | 13.1 (9.8–17.6) | 0.52 (0.46–0.59) | 0.37 (0.31–0.43) | 0.29 (0.24–0.35) |
| **6** | shrub/scrub | fall | 11 | 31.9 (20.6–49.4) | 0.71 (0.62–0.79) | 0.56 (0.46–0.66) | 0.47 (0.38–0.58) |
| **6** | shrub/scrub | spring | 10 | 47.8 (30.9–74.1) | 0.78 (0.7–0.85) | 0.65 (0.55–0.74) | 0.56 (0.47–0.66) |
| **6** | shrub/scrub | summer | 10 | 37 (23.9–57.3) | 0.74 (0.65–0.82) | 0.6 (0.5–0.69) | 0.51 (0.41–0.6) |
| **6** | shrub/scrub | winter | 18 | 48.2 (31.3–74.3) | 0.79 (0.71–0.85) | 0.65 (0.56–0.74) | 0.57 (0.47–0.66) |
| **8** | shrub/scrub | fall | 26 | 13.9 (10.7–18.2) | 0.53 (0.48–0.59) | 0.4 (0.35–0.45) | 0.33 (0.28–0.38) |
| **8** | shrub/scrub | spring | 43 | 20.9 (16.1–27.1) | 0.61 (0.56–0.66) | 0.48 (0.43–0.53) | 0.4 (0.35–0.45) |
| **8** | shrub/scrub | summer | 96 | 16.2 (12.7–20.7) | 0.56 (0.51–0.61) | 0.43 (0.38–0.47) | 0.35 (0.31–0.4) |
| **8** | shrub/scrub | winter | 49 | 21.1 (16.4–27.2) | 0.61 (0.56–0.66) | 0.48 (0.43–0.53) | 0.4 (0.35–0.45) |

All model results are based on the complete meta-dataset of persistence trials from the U.S.
