# Supplementary material for: Game bird carcasses are less persistent than raptor carcasses, but can predict raptor persistence dynamics
Source: PLoS One. 2023 Jan 3;18(1):e0279997. doi: 10.1371/journal.pone.0279997 (PMC9810176; doi:10.1371/journal.pone.0279997)
Supplement: Attachment — Submitted filename: Rebuttal Letter November 2022.docx [file pone.0279997.s012.docx]

November 30, 2022

Editor, PLOS ONE

To Whom it Concerns:

We are pleased to resubmit our manuscript to PLOS ONE entitled: “Game bird carcasses are less persistent than raptor carcasses, but can predict raptor persistence dynamics.” As before, we seek publication of our research article in PLOS ONE.

A copy of the comments resulting from the review of our manuscript by PLOS ONE is provided below. We were pleased to read that the reviewer felt our draft was interesting and well-written. Our responses to their comments are provided below (in red text). Briefly, we agreed with the reviewer that revisions would be helpful in improving readability and comprehension. Most suggested revisions were incorporated; our reasoning behind the few suggestions that were not incorporated is provided.

A rebuttal letter (this document), a marked-up copy of our manuscript that highlights changes made to the previous version, and an unmarked version of our revised paper our included in this submission package. In addition, the supplemental documents and figures that support our manuscript have been included.

No changes to funding, competing interests, or data availability statements are needed. A funding nod was removed from the Acknowledgements section, but this arrangement was already described in our funding statement. Potential copyright issues in the figures (as highlighted by the editor) have been addressed via revisions to the base maps used in those figures.

We hope you agree that our revised manuscript satisfactorily addresses all reviewer comments and meets the high standards of PLOS ONE. We look forward to addressing any further comments from your editors, and to the eventual acceptance and publication of our manuscript.

Best Regards,

Eric Hallingstad (corresponding author)

Address: 415 W 17^th^ St, Suite 200, Cheyenne, WY 82001, USA

Email: [ehallingstad@west-inc.com](mailto:ehallingstad@west-inc.com)

Phone: (509) 386-4616

Reviewers' comments:

Reviewer's Responses to Questions

Comments to the Author

1. Is the manuscript technically sound, and do the data support the conclusions?

The manuscript must describe a technically sound piece of scientific research with data that supports the conclusions. Experiments must have been conducted rigorously, with appropriate controls, replication, and sample sizes. The conclusions must be drawn appropriately based on the data presented.

Reviewer #1: Yes

2. Has the statistical analysis been performed appropriately and rigorously?

Reviewer #1: Yes

3. Have the authors made all data underlying the findings in their manuscript fully available?

The PLOS Data policy requires authors to make all data underlying the findings described in their manuscript fully available without restriction, with rare exception (please refer to the Data Availability Statement in the manuscript PDF file). The data should be provided as part of the manuscript or its supporting information, or deposited to a public repository. For example, in addition to summary statistics, the data points behind means, medians and variance measures should be available. If there are restrictions on publicly sharing data—e.g. participant privacy or use of data from a third party—those must be specified.

Reviewer #1: Yes

4. Is the manuscript presented in an intelligible fashion and written in standard English?

PLOS ONE does not copyedit accepted manuscripts, so the language in submitted articles must be clear, correct, and unambiguous. Any typographical or grammatical errors should be corrected at revision, so please note any specific errors here.

Reviewer #1: Yes

5. Review Comments to the Author

Please use the space provided to explain your answers to the questions above. You may also include additional comments for the author, including concerns about dual publication, research ethics, or publication ethics. (Please upload your review as an attachment if it exceeds 20,000 characters)

Reviewer #1: This is an interesting and well-written study that addresses a topic still understudied: the bias created by used of game bird carcasses (instead of wild specimens) in carcass persistence trials, and their impact on the accuracy of the estimates of bird fatality (more specifically of raptors) in post-construction monitoring studies. Thank you! In addition to a meta-analysis, the authors created a mixed-effects model that predicts raptor persistence as a function of game bird persistence, which will be of great value for monitoring studies (in US) where raptor persistence data is absent.

I have, however, some reservations about the length of the manuscript (> 10,000 words, excluding tables) and the way the results are presented (looking more like a report than a research paper). For instance, if objective 1 is to “compare persistence times and probabilities for large raptor and game bird…”, why are not the results focused on that (3rd section of the “Field trial results”)? Is it necessary to describe separately and in such detail the persistence of raptors and game birds? The same for objective 2: raptor and game bird persistence really need to be described separately, or the focus should be on the “Patterns in raptor and game bird persistence”? We agree with the reviewer that the manuscript was quite long, and we have followed many of their suggestions below to shorten the document. However, in regards to this comment, we are reluctant to shuffle the order in which our results are presented. Before we can compare the persistence metrics between the two bird types, we want to explain how those metrics were estimated—and separate models were needed for each bird type. Thus, we put the model selection material before the comparisons, and elected to retain the organization of the section as well as summary statements on trends observed in model output within each bird type sub-section (only 2-3 paragraphs per sub-section). Retaining the organizational structure helped us maintain parallel structure with the other sections of the manuscript. However, we did follow the reviewer’s suggestion to move Tables 3 and 5 (as well as Tables 4, 6, 7, 8, 9, and 10) to the supplemental materials to improve readability and (hopefully) keep the reader from getting buried too much in the numbers. While comparisons (particularly via the scaling model) were the ultimate objective, our work is the largest analysis of bird persistence data we are aware of, and we feel there is value in presenting the trends observed for each bird type individually. Further comparisons of persistence between bird types are made in the scaling and discussion portions of the ms.

Another problem is the numerous tables included in manuscript, with some of the figures showing the exact same information (for details see my specific comments below). We have followed this suggestion and moved various tables and figures to the supplemental material. See below for additional detail.

In summary, and although presenting the methods and results in a descriptive way is generally regarded as a positive thing, here it makes the manuscript too long and hard to read, which ultimately diverts the readers’ attention from the results obtained for the 3 objectives set for the study.

Specific comments:

L. 35 – Unless you have read the paper, it’s not clear to what meta-dataset you are referring to. We are restricted by the word count limit of the abstract, but have added a brief description.

L. 63 – I would say that it the opposite: “if the bias parameters are high”, meaning if the number of missed carcasses (by imperfect detection or removal), the no. of carcasses that fall outside or the no. of unsearched turbines are high, then the difference between the no. of carcasses found and the no. of fatalities estimated is greater. It would be “low” only if it had been previously explained (L. 60-62) that the bias-correction parameters (by which the observed fatality is divided) are the carcass persistence probability, the detection rate (by the observers), the proportion of carcasses that fall within the search area, and the proportion of searched turbines. Revised for clarity.

L. 90-91 – There has not been a through investigation of persistence data from raptors only, or game vs. wild specimens… But for birds in general there are several studies that have compiled and investigated persistence data from more than a single site, or a small number of sites (e.g., Barrientos et al 2018; Smallwood 2013, doi:10.1002/wsb.260; Bispo et al. 2013, doi: 10.1007/s10651-012-0212-5). Revised for clarity, citations added—including a very recent 2022 paper.

Figure 1 and 3 - Image resolution isn't high enough (it looks pixelated). We think this is an artifact of how the pdf of the draft manuscript was created by the PLOS system. When the tif’s are downloaded using the links in the pdf, the quality appears high. Please let us know if we need to provide higher resolution images for the online version.

L. 176 – The use of the word “fresh” here may be misleading since all carcasses were frozen and then thawed before the trials. Agree, deleted.

L. 200-202 / 207-209 – Include reference or explain why this threshold was used. Citation added.

L. 221 – Not sure why the data from cameratraps had to be “consistent with the in-person checks”, if the R-package “GenEst” was used (see comment to L. 261-276). GenEst can accommodate data both from weekly visits (in-person checks) and from smaller time intervals (cameratraps), as long as a “LastPresent” time and “FirstAbsent” time if provided for each carcass. True. Phrase removed.

L. 257 – Replace “or” by “of”. Done.

L. 261-276 / 316-330 – Were theses analyses done using the GenEst R-package [33]? It is not clear since Ref. 31 to 34 are always cited together, while for the 3rd analysis (“scaling game bird persistence”) it is clearly stated. Yes, clarification added.

L. 378 – Start of stage 2? Yes, clarification added.

L. 396-397 - This last step of stage 2 is not represented in Figure 3, is it? True. Reference to Fig 3 removed, validation segue text added.

Tables 3, 5, 7 and 8 – To reduce the extended number of tables in the MS, please consider the possibility of moving these 4 tables to Supplementary material, as the best supported models for raptor and game persistence are stated in the text, both in objective 1 and 2. Great suggestion. Done.

Table 4 and 6 / Figure 4 to 7 – These tables and figures show the exact same information, right? I would suggest moving Tables 4 and 6 to Supplementary material, with the estimated values still being available for those who may need it. Again, great suggestion. Done.

Tables 9 and 10 – These tables are too long and, consequently, difficult to read… Please considerer moving them to Supplementary material, and better explaining (L. 582-87) why you used the 30-day interval as a showcase your overall results (Fig. 10). Tables 9 and 10 moved to supplemental material. 30-day interval highlighted as most common interval we anticipate in eagle fatality monitoring studies.

L. 522 – I suggest turning this section into a 2nd-level heading, for a better correspondence between the three study objectives (established in the Introduction section) and the structure of the Results section. Done.

L. 763-766 – The persistence of road-kills is highly influenced by traffic; thus, it is not comparable with carcass persistence dynamics at WEFs and power lines, where removal occurs mostly due to scavenging or decomposition. While we agree that vehicles play a role in removal of carcasses from roadways, the authors of the cited paper found that persistence was actually shorter on less-traveled roads. This suggests removal of small carcasses through other means (e.g., scavenging) was prevalent, and the authors explicitly state this likelihood (which we refer to in our ms). Therefore, we kept the statement and supporting citation in our ms.

6. PLOS authors have the option to publish the peer review history of their article (what does this mean?). If published, this will include your full peer review and any attached files.

If you choose “no”, your identity will remain anonymous but your review may still be made public.

Do you want your identity to be public for this peer review? For information about this choice, including consent withdrawal, please see our Privacy Policy.

Reviewer #1: No
